# A phase I pharmacokinetics study comparing PF-06439535 (a potential biosimilar) with bevacizumab in healthy male volunteers

**DOI:** 10.1007/s00280-016-3001-2

**Published:** 2016-03-16

**Authors:** Beverly Knight, Danielle Rassam, Shanmei Liao, Reginald Ewesuedo

**Affiliations:** Clinical Pharmacology, Pfizer Inc, 10555 Science Center Drive, San Diego, CA 92121 USA; Biosimilars Clinical Development, Pfizer Inc, San Diego, CA USA; Clinical Statistics, Pfizer Inc, Shanghai, People’s Republic of China; Biosimilars Clinical Development, Pfizer Inc, Cambridge, MA USA

**Keywords:** PF-06439535, Bevacizumab, Biosimilar, Immunogenicity, Pharmacokinetics

## Abstract

**Purpose:**

This study compared the pharmacokinetics of PF-06439535, a potential bevacizumab biosimilar, to bevacizumab sourced from the European Union (bevacizumab-EU) and USA (bevacizumab-US), and of bevacizumab-EU to bevacizumab-US.

**Methods:**

In this double-blind study, 102 healthy males, aged 21–55 years, were randomized 1:1:1 to receive a single 5 mg/kg intravenous dose of PF-06439535, bevacizumab-EU, or bevacizumab-US. Pharmacokinetic assessments were conducted for 71 days, with additional safety and immunogenicity assessments until day 100. Pharmacokinetic similarity was achieved if 90 % confidence intervals (CIs) for the test-to-reference ratios of the maximum serum concentration (*C*_max_), area under the serum concentration–time curve from zero to infinity (AUC_0–∞_), and from zero to time of last quantifiable concentration (AUC_0–*t*_) were within the 80.00–125.00 % bioequivalence acceptance window.

**Results:**

The three study drugs exhibited similar pharmacokinetic properties. For the comparisons of PF-06439535 to bevacizumab-EU or bevacizumab-US, and of bevacizumab-EU to bevacizumab-US, the 90 % CIs for the ratios of *C*_max_, AUC_0–*t*_, and AUC_0–∞_ were all within 80.00–125.00 %. Two, one, and two subjects treated with PF-06439535, bevacizumab-EU, and bevacizumab-US, respectively, tested positive for antidrug antibodies, none of whom tested positive for neutralizing antibodies. Treatment-related adverse events were reported in 15.2, 25.7, and 18.2 % of subjects in the PF-06439535, bevacizumab-EU, and bevacizumab-US treatment arms, respectively.

**Conclusions:**

This study demonstrated the pharmacokinetic similarity of PF-06439535 to both bevacizumab-EU and bevacizumab-US, and of bevacizumab-EU to bevacizumab-US. The safety profile (including immunogenicity) was similar in the three treatment groups, with no significant safety findings reported.

**Electronic supplementary material:**

The online version of this article (doi:10.1007/s00280-016-3001-2) contains supplementary material, which is available to authorized users.

## Introduction

Biologics are large and structurally complex molecules produced in living cells and therefore cannot be exactly duplicated. The term “biosimilars” is used to describe biologics that are highly similar but not identical to already licensed or approved biologics in terms of safety, purity, and potency [1–3]. Biosimilars have the potential to increase global patient access to biologics, which may improve the overall health outcomes. Unlike small-molecule drugs that are chemically synthesized and can be copied exactly, the manufacturing process of biosimilars is complex; even minor structural differences may have a significant impact on the efficacy and safety of a biosimilar [2, 3]. Regulatory approval of biosimilars is based on the totality of the evidence generated from a comprehensive comparison of the proposed biosimilar and the reference product, which include structure and function characterization, nonclinical evaluation, human pharmacokinetics (PK) and pharmacodynamics (PD), and clinical safety (including immunogenicity) and efficacy data [1–3].

Bevacizumab (Avastin^®^) is recombinant humanized monoclonal immunoglobulin G1 antibody that inhibits angiogenesis by binding to the human vascular endothelial growth factor (VEGF) and inhibiting its biologic activity [4]. Bevacizumab is approved in the USA and Europe for the treatment of patients with non-small cell lung cancer (NSCLC), metastatic colorectal cancer, metastatic renal cell cancer, and cervical, platinum-resistant recurrent epithelial ovarian, fallopian tube, and primary peritoneal cancers. Additionally, it is approved for the treatment of patients with glioblastoma multiforme in the USA and for metastatic breast cancer in Europe. The specific indication may vary in other regions/countries, and additional indications are being pursued by the manufacturer [4].

PF-06439535 is being developed as a potential biosimilar to bevacizumab. PF-06439535 has an identical primary structure, and similar posttranslational modifications, biochemical properties, and biologic function as bevacizumab reference products [5]. A nonclinical toxicity study in cynomolgus monkeys has also demonstrated the similarity of PF-06439535 with bevacizumab [6]. The results from the analytical and nonclinical in vivo studies supported the initiation of the clinical development.

PK studies in humans comparing a proposed biosimilar with the reference product are essential for the demonstration of biosimilarity and should be conducted in a population using dose(s), and route of administration that are adequately sensitive to detect differences in PK profiles [1–3]. Here we report the results from a single-dose PK study in healthy male volunteers. Assessment of PK using a single-dose study design in healthy volunteers was expected to be the most sensitive setting possible to detect intrinsic differences in PK between PF-06439535 and bevacizumab. The study design avoided factors that could confound the interpretation of PK results, such as the potential for variability associated with a multidose, multicenter regimen, varying tumor burden and complications inherent with disease indications, comorbidities, and concomitant therapies and medications. The selection of (only) male subjects for this study was based on the documented influence of gender on bevacizumab PK: Clearance is approximately 26 % higher in males compared with females [4].

Therapeutic doses in the prescribing label of the reference products ranged from 5 mg/kg every 2 weeks to 15 mg/kg every 3 weeks [4, 7]. The dosage used in this study, 5 mg/kg, was selected based on a prior study (data on file) to balance safety considerations in healthy subjects, the need to capture the full PK profile of bevacizumab for area under the serum concentration–time curve (AUC) estimation, and the need to sensitively compare PK among the study drugs.

The primary objective of this phase I study was to compare the PK of PF-06439535 versus bevacizumab sourced from the European Union (bevacizumab-EU; Avastin^®^, F. Hoffman-La Roche, Basel, Switzerland) [7] and USA (bevacizumab-US; Avastin^®^, Genentech Inc, South San Francisco, CA, USA) [4] and to compare the PK of bevacizumab-EU with bevacizumab-US. This study also evaluated safety, tolerability, and immunogenicity following a single dose of PF-06439535, bevacizumab-EU, or bevacizumab-US.

## Methods

### Study population

Subjects included in this study were healthy males 21–55 years of age with body mass index of 18.0–30.5 kg/m^2^ and a total body weight >50 kg. Subjects were required to have adequate organ function (excluding subjects who received blood transfusions) according to the following laboratory values: bone marrow function (absolute neutrophil count ≥1500/mm^3^ and platelet count ≥100,000/mm^3^), adequate liver function [alanine aminotransferase (ALT) ≤3 × upper limit normal (ULN) and alkaline phosphatase ≤2 × ULN, total bilirubin ≤1.5 mg/dL], and adequate renal function (blood urea nitrogen ≤1.5 × institutional normal and creatinine <1.5 mg/dL) upon study entry.

Key exclusion criteria included evidence or history of clinically significant disease, previous history of cancer other than adequately treated basal cell or squamous cell carcinoma of the skin, hypertension (defined as blood pressure ≥140/90 mmHg), and previous treatment with an anti-VEGF antibody or any other antibody or protein targeting the VEGF receptor.

### Study design

This phase I, double-blind, randomized, parallel-group, single-dose, three-arm study was conducted in one center in the USA between January 24, 2014, and August 5, 2014 (ClinicalTrials.gov identifier NCT02031991, Supplementary Fig. S1). The final protocol, any amendments, and informed consent documentation were reviewed and approved by the Institutional Review Board (Aspire IRB, Santee, CA, USA). The study was conducted in compliance with the Declaration of Helsinki, all International Conference on Harmonization Good Clinical Practice Guidelines, and local regulatory requirements. All subjects provided informed consent before any screening procedures were done.

After a screening visit that occurred within 28 days prior to dosing, eligible subjects were admitted to the clinical research unit on the day before dosing. Following an overnight fast of at least 8 h, subjects were randomized to receive a single 5 mg/kg intravenous dose of PF-06439535, bevacizumab-EU, or bevacizumab-US in a 1:1:1 ratio according to a computer-generated randomization schedule. Subjects were discharged after 8 days and returned to the clinical research unit on an outpatient basis for additional analyses on days 15, 22, 29, 43, 57, 64, 71, and (optional) 100. Blood samples for the primary PK analysis were collected prior to treatment and at specified time points through day 71; an additional serum concentration sample was collected on day 100 to support the antidrug antibodies (ADA) analysis. Safety assessments (including immunogenicity) were evaluated for 71 days with an additional, optional day 100 visit to assess immunogenicity in the absence of interfering drug levels.

### Pharmacokinetic evaluations

Blood samples for PK evaluation were collected within 1 h prior to initiation of bevacizumab infusion (predose), within 5 min prior to end of infusion (90 min after start of infusion), and at 4, 24, 48, 96, 168, 336, 504, 672, 1008, 1344, 1512, and 1680 h after start of infusion. Blood samples were centrifuged at 1500–1700*g* for approximately 15–20 min in a refrigerated centrifuge. The plasma was stored at −70 °C until analysis.

Serum concentrations of PF-06439535, bevacizumab-EU, and bevacizumab-US were analyzed using a validated, sensitive, and specific enzyme-linked immunosorbent assay at QPS, LLC (Newark, DE, USA). The lower limit of quantification (LLOQ) was 250 ng/mL; samples below the LLOQ were set to 0 for the PK analysis. The inter-run assay accuracy, expressed as percent relative error for quality control samples, ranged from −3.3 to −1.8 %. The assay precision, expressed as the inter-run coefficients of variation of the estimated concentrations of quality control samples, was <15.9 %.

The PK parameters were calculated for each eligible subject using standard noncompartmental analysis of concentration–time data and included maximum observed serum concentration (*C*_max_), AUC from zero to the time of the last quantifiable concentration (AUC_0–*t*_), AUC from zero extrapolated to infinity (AUC_0–∞_), clearance (CL), volume of distribution at steady state (*V*_ss_), and terminal half-life (*t*_½_). The PK analysis used actual sample collection times. PK parameters were calculated using an internally validated software system, eNCA (version 2.2.4).

### Immunogenicity evaluations

Blood samples to detect ADA and neutralizing antibodies (NAb) were collected at 0, 15, 29, 57, 71, and (optional) 100 days post-dose. An additional sample for drug concentration was taken on day 100 to facilitate the immunogenicity assessment at 100 days post-dose. ADA samples were analyzed at QPS, LLC using two validated, semiquantitative electrochemiluminescent assays: one to detect antibodies against PF-06439535 and one to detect antibodies against bevacizumab. Samples testing positive for ADA were further tested for the presence or absence of neutralizing anti-PF-06439535 or anti-bevacizumab antibodies, using two validated semiquantitative electrochemiluminescent NAb assays.

### Safety evaluations

All observed or patient-reported adverse events (AEs), graded by the National Cancer Institute Common Terminology Criteria for Adverse Events, version 4.03, were assessed for severity and relationship to the study drug treatment. Subjects who had an unresolved AE were followed up until the AE or its sequelae resolved or stabilized per the investigator’s assessment. Other safety assessments included laboratory tests (hematology, chemistry, and urinalysis), physical examinations, vital signs (supine blood pressure, pulse rate, respiratory rate, and body temperature), electrocardiograms, telemetry, and pulse oximetry.

### Statistical analyses

PK similarity was achieved if 90 % confidence intervals (CIs) for the test-to-reference ratios of *C*_max_, AUC_0–*t*_, and AUC_0–∞_ fell within the 80.00–125.00 % acceptance criteria for the following comparisons: PF-06439535 versus bevacizumab-EU, PF-06439535 versus bevacizumab-US, and bevacizumab-EU versus bevacizumab-US. Ninety percent CIs for the test-to-reference ratios for AUC_0–∞_, AUC_0–*t*_, and *C*_max_ were constructed on a log scale using two one-sided test (TOST) procedure.

Based on a prior PK study (data on file), a conservative estimate of percent coefficient of variation (%CV) for AUC_0–∞_ of 21 % was used. A sample size of 29 subjects per arm provided at least 85 % power for the two comparisons in AUC_0–∞_ for the similarity objective if the true ratio of AUC_0–∞_ values was equal to 1.05 or less. Since AUC_0–∞_ and AUC_0–*t*_ were highly correlated, the power for similarity in AUC_0–*t*_ was about the same as that for AUC_0–∞_. Similarly, if the observed %CV for *C*_max_ was 21 %, a sample size of 29 subjects per arm provided at least 85 % power for the two comparisons to achieve similarity in *C*_max_ for the similarity objective if the true ratio of *C*_max_ values was equal to 1.05 or less.

Overall, a sample size of 29 subjects per arm was selected to provide no less than 70 % power to demonstrate similarity for all three pair-wise comparisons. To account for a nonevaluable rate of approximately 10 %, the total sample size was increased to approximately 96 subjects, with 32 subjects per arm.

The per-protocol analysis set, which included all randomized subjects who received the full dose of the assigned study medication and who did not have major protocol deviations, was used as the population for PK analysis. The safety analysis set included all enrolled subjects who received the study medication.

## Results

### Subjects

**Fig. 1:**
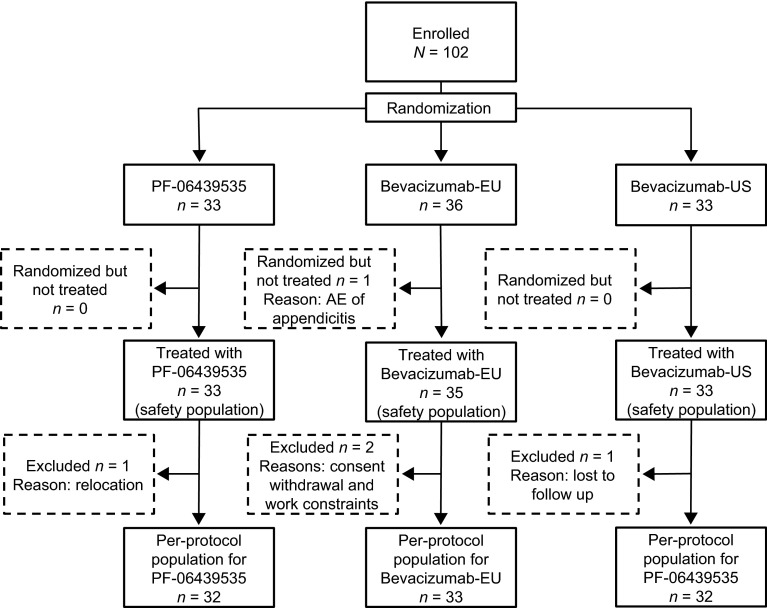
Subject disposition. *AE* adverse event; *PK* pharmacokinetics

Of a total 102 subjects enrolled and assigned to study treatment, 101 received the assigned study drug (PF-06439535, *n* = 33; bevacizumab-EU, *n* = 35; bevacizumab-US, *n* = 33) and constituted the safety analysis set (Fig. 1). Four subjects (PF-06439535, *n* = 1; bevacizumab-EU, *n* = 2; bevacizumab-US, *n* = 1) were excluded from the primary PK analysis due to premature study withdrawal not related to study drug. The final per-protocol population used in the PK analysis consisted of 97 subjects. The demographic and baseline characteristics in the per-protocol population were comparable among the three treatment groups (Table 1).

**Table 1:** Demographic and baseline characteristics (per-protocol population)

	PF-06439535	Bevacizumab-EU	Bevacizumab-US
	*n* = 32^a^	*n* = 33^a^	*n* = 32^a^
Age (years)			
Mean (SD)	37.6 (±8.7)	39.1 (±11.0)	36.0 (±8.7)
Range	22–53	21–55	21–50
Race, *n*			
White	26	26	24
Black	6	7	7
Asian	0	0	1
Ethnicity, *n*			
Hispanic/Latino	29	29	25
Not Hispanic/Latino	3	4	7
Weight (kg)			
Mean (SD)	79.3 (±10.6)	78.6 (±9.4)	77.9 (±11.7)
Range	55.4–99.1	55.5–92.1	51.5–102.0
Body mass index (kg/m^2^)			
Mean (SD)	26.5 (±2.9)	26.4 (±2.8)	25.5 (±3.0)
Range	19.9–30.4	19.2–30.5	18.0–30.1
Height (cm)			
Mean (SD)	172.9 (±7.0)	172.6 (±5.6)	174.6 (±6.6)
Range	160.0–186.0	157.0–185.0	162.0–187.0

*SD* standard deviation

^a^Number of evaluable subjects

### Pharmacokinetic evaluations

**Fig. 2:**
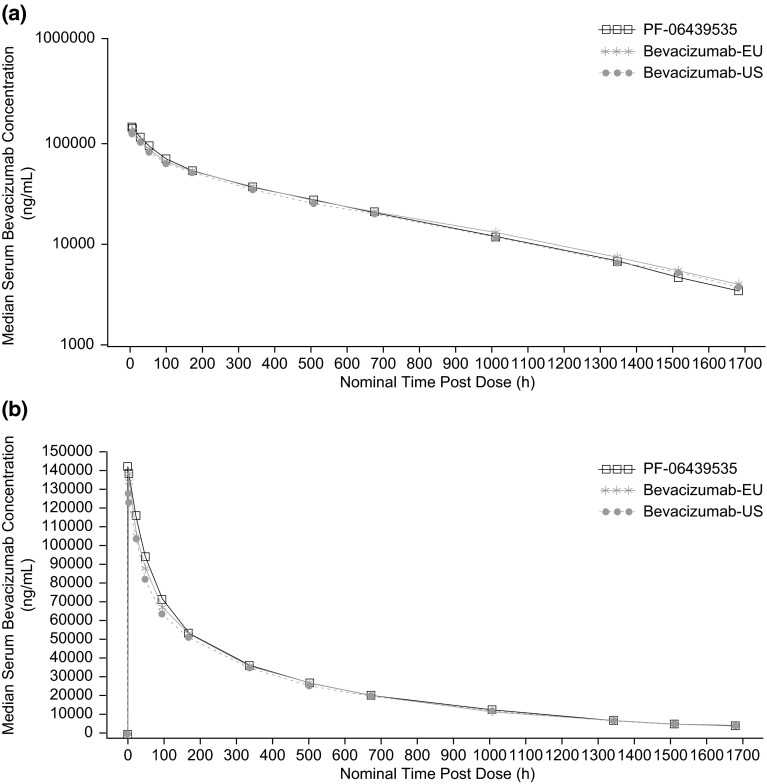
Median serum concentration–time profiles following a single 5 mg/kg intravenous dose in healthy subjects. **a** Semi-logarithmic scale, **b** linear scale

The three study drugs exhibited a similar median serum concentration–time profile characterized by a rapid decrease in serum drug concentration immediately following the end of infusion, followed by a slow elimination phase (Fig. 2). Consistent with the mean concentration–time profiles, the mean *C*_max_, AUC_0–*t*_, and AUC_0–∞_ estimates and inter-subject variability for each PK parameter were similar across the three study drugs, with coefficients of variation values of 14–15 % for *C*_max_, 12–16 % for AUC_0–*t*_, and 13–19 % for AUC_0–∞_ (Table 2, Supplementary Fig. S2). For the comparisons of PF-06439535 to bevacizumab-EU or bevacizumab-US, and for bevacizumab-EU to bevacizumab-US, the 90 % CIs for the test-to-reference ratios of *C*_max_, AUC_0–*t*_, and AUC_0–∞_ were all within the bioequivalence window of 80.00–125.00 % (Table 3).

**Table 2:** Mean (±SD) pharmacokinetic parameter estimates of PF-06439535, bevacizumab-EU, and bevacizumab-US

Parameters, units	PF-06439535 *n* = 32	Bevacizumab-EU *n* = 33	Bevacizumab-US *n* = 32
*C*_max_ (µg/mL)	142.9 ± 20.3	137.0 ± 20.5	130.0 ± 18.2
AUC_0–*t*_ (µg·h/mL)^a^	40,840 ± 6411	41,010 ± 6711	38,920 ± 4566
AUC_0–∞_ (µg·h/mL)	43,080 ± 7103	43,830 ± 8326	41,450 ± 5350
CL (mL/h/kg)	0.119 ± 0.021	0.117 ± 0.022	0.122 ± 0.016
*V*_ss_ (mL/kg)	62.4 ± 10.6	64.9 ± 9.6	67.7 ± 7.7
*t*_½_ (h)	397 ± 63	417 ± 90	413 ± 57

*AUC*_*0*–*∞*_ area under the serum concentration–time curve from zero extrapolated to infinity, *AUC*_*0*–*t*_ area under the serum concentration–time curve from zero to the time of the last quantifiable concentration, *CL* clearance, *C*_*max*_ maximum observed serum concentration, *SD* standard deviation, *t*_*½*_ terminal half-life, *V*_*ss*_ volume of distribution at steady state

^a^AUC_0–*t*_ was ≥80 % of the corresponding AUC_0–∞_ in 97 (100 %) pharmacokinetic eligible subjects

**Table 3:** Statistical comparison of pharmacokinetic exposure parameters

Test	Reference	Parameter^a^	Adjusted geometric means			
			Test	Reference	Ratio (%)^b^	90 % CI (%)
PF-06439535	Bevacizumab-EU	*C*_max_	141.5	135.5	104.42	98.36–110.84
		AUC_0–*t*_	40,330	40,490	99.62	93.69–105.93
		AUC_0–∞_	42,490	43,100	98.58	92.16–105.44
PF-06439535	Bevacizumab-US	*C*_max_	141.5	128.9	109.79	103.38–116.60
		AUC_0–*t*_	40,330	38,660	104.32	98.06–110.97
		AUC_0–∞_	42,490	41,120	103.33	96.55–110.58
Bevacizumab-EU	Bevacizumab-US	*C*_max_	135.5	128.9	105.15	99.05–111.62
		AUC_0–*t*_	40,490	38,660	104.71	98.48–111.34
		AUC_0–∞_	43,100	41,120	104.82	98.00–112.12

*AUC*_*0*–*∞*_ area under the serum concentration–time curve from zero extrapolated to infinity, *AUC*_*0*–*t*_ area under the serum concentration–time curve from time 0 to the time of the last quantifiable concentration, *CI* confidence interval, *C*_*max*_ the maximum observed serum concentration

^a^ *C*_max_, AUC_0–*t*_, and AUC_0–∞_ units are measured in μg/mL, μg·h/mL, and μg·h/mL, respectively

^b^Test-to-reference ratio of adjusted geometric means

### Immunogenicity evaluations

Of the 101 subjects in the safety population, 94 completed ADA assessments at specified visits through day 71, and 91 subjects completed ADA assessment through day 100. Overall, the three treatment groups had similar ADA profiles. When PF-06439535 was used as the capture reagent, one subject treated with bevacizumab-EU tested positive for ADA at baseline. Five (5.0 %) subjects tested positive at one or more time point through day 100: *n* = 2 (6.1 %), *n* = 1 (2.9 %), and *n* = 2 (6.1 %) of total number of subjects administered PF-06439535, bevacizumab-EU, and bevacizumab-US, respectively. Of the five subjects (total of seven samples through day 100) who tested positive for ADA post-dose, three (PF-06439535, *n* = 2; bevacizumab-US, *n* = 1) did not have detectable levels of ADA until day 71. The other two subjects with treatment-emergent ADA had more than one sample that tested positive: One subject treated with bevacizumab-EU tested positive at predose and days 71 and 100, and one subject treated with bevacizumab-US tested positive at days 57 and 71.

The samples were also tested with a second ADA assay using bevacizumab-EU as the capture reagent. Only one sample tested positive on day 71 with the second assay, whereas the remaining samples that were positive in the first assay (all of which had titers close to the assay cut-point of 2.29) tested negative with the second assay.

The eight samples that tested positive for ADA were further tested for NAb; none of these samples tested positive for NAb. However, it should be noted that drug serum concentrations were high (range 0.29–205 µg/mL) relative to the drug tolerance of the NAb assay (range 0.5–10 µg/mL).

### Safety evaluations

No deaths or discontinuations due to AEs occurred in this study. Among the 101 subjects who received study drug, 55 (54.5 %) experienced treatment-emergent AEs and 20 of these subjects experienced 31 treatment-related AEs [*n* = 5 (15.2 %), 9 (25.7 %), and 6 (18.2 %) in the PF-06439535, bevacizumab-EU, and bevacizumab-US treatment arms, respectively]. Most AEs were grade 1 or 2, except for one grade 3 AE of musculoskeletal pain and one grade 4 AE of concussion experienced by one subject (Table 4); both the grade 3 and grade 4 AEs occurred due to a motor vehicle accident (subject was a passenger) and were resolved by the end of study. The AE of concussion was reported as a serious AE. Another serious AE of appendicitis was reported in one subject prior to randomization to study treatment.

**Table 4:** Treatment-emergent adverse events occurring in two or more subjects in any treatment group: all-causality (safety analysis population)

MedDRA preferred term, *n* (%)	PF-06439535 *n* = 33^a^	Bevacizumab-EU *n* = 35^a^	Bevacizumab-US *n* = 33^a^
Subjects with AEs	16 (48.5)	22 (62.9)	17 (51.5)
Subjects with SAEs	0	1 (2.9)	0
Subjects with grade 3 or grade 4 AEs^b^	0	1 (2.9)	0
Upper respiratory tract infection	4 (12.1)	6 (17.1)	4 (12.1)
Headache	2 (6.1)	3 (8.6)	3 (9.1)
Dyspepsia	2 (6.1)	3 (8.6)	1 (3.0)
Myalgia	1 (3.0)	2 (5.7)	2 (6.1)
Diarrhea	2 (6.1)	1 (2.9)	1 (3.0)
Tooth abscess	2 (6.1)	1 (2.9)	0
Musculoskeletal pain	1 (3.0)	2 (5.7)	0
Rash macular	1 (3.0)	2 (5.7)	0

*AE* adverse event, *MedDRA* Medical Dictionary for Regulatory Activities, version 17.0, *SAE* serious adverse event

^a^Number of subjects in the treatment group

^b^No subject had grade 5 AEs

Laboratory results, physical examination findings, vital signs, and electrocardiogram values were unremarkable, with no safety issues identified and with no clinically meaningful differences among the three treatment arms.

## Discussion

PF-06439535 is being developed as a potential biosimilar to bevacizumab and has been shown to have identical primary structure and similar posttranslational modifications, biochemical properties, and biologic function as the reference product bevacizumab [5, 6]. The primary objective of the current phase I study was to demonstrate PK similarity of PF-06439535 to bevacizumab-EU and bevacizumab-US and of bevacizumab-EU to bevacizumab-US in healthy volunteers. The 90 % CIs for the test-to-reference ratios of the calculated exposure parameters (*C*_max_ and AUC) were within the predefined bioequivalence acceptance range of 80.00–125.00 % for the comparison of PF-06439535 to bevacizumab-EU and bevacizumab-US and of bevacizumab-EU to bevacizumab-US, demonstrating PK similarity among the three products. The demonstration of PK similarity between the two licensed bevacizumab products sourced from the European Union and the USA provides justification for the use of only one of these reference products in future comparative clinical studies in patients.

As with all biologic agents, immune responses against bevacizumab may develop. In this study, the three treatment groups had comparable ADA profiles with no NAb detected in any of the ADA-positive serum samples. The low incidence of ADA observed in all three treatment groups was consistent with the published immunogenicity results in patients treated with the reference product [4]. These data emphasize the low immunogenicity of bevacizumab and PF-06439535.

All three study drugs showed comparable safety profiles with no clinically meaningful differences observed among PF-06439535, bevacizumab-EU, and bevacizumab-US. The most commonly reported AE, occurring in more than 10 % of participants, was upper respiratory tract infection, which was distributed evenly among the three treatment groups. Overall laboratory results and other safety measures were unremarkable, with no safety issues identified.

## Conclusions

The present study demonstrates the PK similarity of PF-06439535 to both bevacizumab-EU and bevacizumab-US, and of bevacizumab-EU to bevacizumab-US. The three treatment groups had a similar ADA profile with no NAb detected. All three study drugs showed comparable safety profiles, with no significant AEs or other safety findings reported. These data support the continued development of PF-06439535 as a proposed biosimilar to bevacizumab. An ongoing phase III, multinational, double-blind, randomized, parallel-group clinical trial (ClinicalTrials.gov, NCT02364999) in previously untreated patients with advanced nonsquamous NSCLC is evaluating the efficacy, safety, and immunogenicity of PF-06439535 versus bevacizumab-EU in combination with paclitaxel and carboplatin.

## Electronic supplementary material

Below is the link to the electronic supplementary material.

Fig. S1 Study design. ^a^ Hours at which samples were collected for ADA. Samples for PK were collected at all times shown. *ADA* antidrug antibody; *AE* adverse event, *PK* pharmacokinetics. (EPS 1968 kb)

Fig. S2 Individual and geometric mean *C*_max_, AUC_0-*t*_, and AUC_0-∞_ for PF-06439535, bevacizumab-EU, and bevacizumab-US. **(a)** *C*_max_; **(b)** AUC_0-*t*_; and **(c)** AUC_0**-∞**_. Stars (★) represent geometric mean and circles (o) represent individual subject values. Box plot provides median and 25 %/75 % quartiles with whiskers to the last point within 1.5 × inter-quartile range. *AUC*_*0*-*∞*_ area under the serum concentration-time profile from zero extrapolated to infinite time; *AUC*_*0*-*t*_ area under the serum concentration-time profile from zero to the time of the last quantifiable concentration; *C*_*max*_ maximum observed serum concentration. (EPS 2415 kb)

## References

[1] European Medicines Agency (2014) Guideline on similar biological medicinal products. http://www.ema.europa.eu/docs/en_GB/document_library/Scientific_guideline/2014/10/WC500176768.pdf. Accessed 25 Aug 2015

[2] US Food and Drug Administration (2015) Scientific considerations in demonstrating biosimilarity to a reference product. http://www.fda.gov/downloads/drugs/guidancecomplianceregulatoryinformation/guidances/ucm291128.pdf. Accessed 1 May 2015

[3] World Health Organization, Expert Committee on Biological Standardization (2009) Guidelines on evaluation of similar biotherapeutic products (SBPs). http://www.who.int/biologicals/areas/biological_therapeutics/BIOTHERAPEUTICS_FOR_WEB_22APRIL2010.pdf. Accessed 4 Feb 2015

[4] Genentech Inc (2015) Avastin^®^ (bevacizumab) prescribing information. http://www.gene.com/download/pdf/avastin_prescribing.pdf. Accessed 20 May 2015

[5] Grunder B, Costigan L, Johnson K, Kneeland T, Cirelli D, Dufield R, Kitchen R, Porter T, Rouse J, Rule K (2014) Characterization and similarity assessment of bevacizumab and a proposed biosimilar. Presented at: American Association of Pharmaceutical Scientists (AAPS) Annual Meeting and Exhibition, San Diego, CA

[6] Rule K, Peraza M, Shiue M, Finch G, Thibault S, Rosenberg JA, Leach MW (2015) Nonclinical development of PF-06439535, a potential biosimilar to bevacizumab. Presented at: IASLC 16th World Conference on Lung Cancer (WCLC 2015), Denver, CO

[7] Socinski MA, Curigliano G, Jacobs I, Gumbiner B, MacDonald J, Thomas D (2015). Clinical considerations for the development of biosimilars in oncology. MAbs.

